# The effect of school summer holidays on inequalities in children and young people’s mental health and cognitive ability in the UK using data from the millennium cohort study

**DOI:** 10.1186/s12889-022-12540-2

**Published:** 2022-01-22

**Authors:** Theocharis Kromydas, Mhairi Campbell, Stephanie Chambers, Michele Hilton Boon, Anna Pearce, Valerie Wells, Peter Craig

**Affiliations:** 1 MRC/CSO Social and Public Health Sciences Unit, Institute of Health and Wellbeing, University of Glasgow, Berkeley Square, 99 Berkeley Street, Glasgow, G3 7HR UK; 2 School of Social and Political Sciences, University of Glasgow, Glasgow, UK

**Keywords:** School closures, Mental health, Cognitive ability, Inequalities, COVID-19

## Abstract

**Background:**

Summer learning loss has been the subject of longstanding concern among researchers, the public and policy makers. The aim of the current research was to investigate inequality changes in children’s mental health and cognitive ability across the summer holidays.

**Methods:**

We conducted linear and logistic regression analysis of mental health (borderline-abnormal total difficulty and prosocial scores on the strengths and difficulties questionnaire (SDQ)) and verbal cognitive ability (reading, verbal reasoning or vocabulary) at ages 7, 11 and 14, comparing UK Millennium Cohort Study members who were interviewed before and after the school summer holidays. Inequalities were assessed by including interaction terms in the outcome models between a discrete binary variable with values representing time periods and maternal academic qualifications. Coefficients of the interaction terms were interpreted as changes from the pre- to post-holiday period in the extent of inequality in the outcome between participants whose mothers had high or low educational qualifications. Separate models were fitted for each age group and outcome. We used inverse probability weights to allow for differences in the characteristics of cohort members assessed before and after the summer holidays.

**Results:**

Mental health (borderline/abnormal SDQ total and prosocial scores) at ages 7 and 14 worsened and verbal cognitive ability scores at age 7 were lower among those surveyed after the summer holidays. Mental health inequalities were larger after the holidays at age 7 ([OR = 1.4; 95%CI (0.6, 3.2) and 14: [OR = 1.5; 95%CI (0.7, 3.2)], but changed little at age 11 (OR = 0.9; 95%CI (0.4, 2.6)]. There were differences in pro-social behaviours among those surveyed before/after the school holidays at age 14 [OR = 1.2; 95%CI (0.5, 3.5)] but not at age 7 or 11. There was little change in inequalities in verbal cognitive ability scores over the school holidays [Age 7: b = 1.3; 95%CI (− 3.3, 6.0); Age 11: b = − 0.7; 95%CI (− 4.3, 2.8); Age 14: b = − 0.3; 95%CI (− 1.0, 0.4)].

**Conclusion:**

We found inequalities in mental health and cognitive ability according to maternal education, and some evidence or worsening mental health and mental health inequalities across school summer holidays. We found little evidence of widening inequalities in verbal cognitive ability. Widespread school closures during the COVID-19 restrictions have prompted concerns that prolonged closures may widen health and educational inequalities. Management of school closures should focus on preventing or mitigating inequalities that may arise from differences in the support for mental health and learning provided during closures by schools serving more or less disadvantaged children.

**Supplementary Information:**

The online version contains supplementary material available at 10.1186/s12889-022-12540-2.

## Introduction

Socio-economic differences in summer learning loss have been the subject of widespread concern among researchers, the public and policy makers since a landmark US study [1] found that a large fraction of total inequality in educational attainment between children from more and less affluent backgrounds was attributable to differences that emerge over the school summer holidays. The existence and seriousness of the problem appeared to be confirmed by a systematic review published in 1996 [2], but recent research has suggested that the apparent widening of inequalities is an artefact of the way children’s ability is measured and analysed [3, 4]. Reanalyses of previously published data and analyses of other datasets indicate a much smaller effect or no effect of summer learning loss on socio-economic inequalities in attainment [5–7]. Since the review by Cooper et al. [2], analysis of socio-economic status and reading ability has not identified consistent evidence of inequalities between advantaged and disadvantaged students. While negative effects have been found for students with lower socio-economic status [8–16], other studies found negative effects for higher socio-economic students [17, 18]. Many recent studies report conflicting results, such as different effects for different school grades, datasets, measurement scaling, or analysis models used [5–7, 19–22], or no difference between socio-economic groups [21, 23–26].

Despite these recent findings, concerns persist over differential learning loss when schools are closed [27]. The National Summer Learning Association asserts on its website that the cumulative effect of summer learning loss is ‘a crisis in the making: by the fifth grade, summer learning loss can leave low-income students two-and-a-half to 3 years behind their peers.’ [28]. In the UK, concern over summer learning loss has been compounded by fears that many children experience ‘holiday hunger’ when schools close for the summer and stop providing free meals [29].

In the debates over whether and how long schools should be closed to help manage the pandemic, references to summer learning loss have been frequent. The finding from a rapid review of evidence by the Education Endowment Foundation [30] that inequalities could widen substantially if schools remained closed for 6 months was widely cited by senior policy makers and politicians in the UK [31–33]. Against this backdrop of continuing debate about the effects of school closures on inequalities in attainment, and their contribution to managing the pandemic, we undertook the first analysis of UK-wide data on changes in socio-economic inequalities in children and young people’s cognitive ability and mental health over the school summer holidays.

## Methods

The UK Millennium Cohort Study is a nationally representative, longitudinal survey of children born in the UK from September 2000 to January 2002 [34]. The survey uses a stratified clustered sampling design to oversample children living in Wales, Scotland and Northern Ireland, disadvantaged areas and, in England, areas with high proportions of ethnic minority groups. We used data gathered when the cohort members were aged 7 (*n* = 13,681), 11 (*n* = 13,112) and 14 (*n* = 11,564), with interviews spread over a year or more [34].

Since our aim is to investigate changes in mental health and verbal cognitive ability across the summer holidays, we restricted our analysis to cohort members surveyed in the 2 months (three in the case of Scotland) before and the 2 months after the summer holidays in each sweep (further detail is provided in the following section). Using the whole sample across the pre- and post-summer holiday months would have confounded the effects of the summer holiday with those of other holiday periods throughout the year, and with the effects of catch-up once cohort members return to school. In addition to the survey weights, we used stabilised inverse probability weighting to correct for the varying composition of the sample in the pre- and post-holiday months.

### School summer holidays

We measure exposure to summer holidays using a binary variable that differentiates between cohort members surveyed in the months preceding the summer holidays (baseline) and the months after the summer holidays. We defined pre- and post-summer holiday groups differently across the four UK countries, to allow for differences in the timings of the start and finish of school summer holidays and to maximise power. We defined pre- and post-holiday months as June–July and September–October for England and Wales (where summer holidays tend to run from mid-July to early September), but as April–June and August–September in Scotland (where summer holidays run from late June to mid-August) and May–June and August–September for Northern Ireland (where summer holidays are throughout July and August). We included the extra month for Scotland because very few interviews are conducted in June (Table A1a).

### Outcomes

Our outcomes of interest are verbal cognitive ability and mental health as represented by socio-emotional well-being. Verbal cognitive ability was measured using the British Ability Scale (BAS) word reading score at age 7; at age 11 we use the BAS verbal abilities scale and at age 14 we use a word activity score based on subsets of the words used in a vocabulary assessment in the 1970 British Cohort Study (BCS70) Age 16 Survey (the words used in the BCS70 assessment are derived from the standardised vocabulary tests devised by the Applied Psychology Unit at the University of Edinburgh in 1976). Although each sweep of the Millennium Cohort Study uses a different measure of verbal cognitive ability, all cohort members within each sweep are tested in the same way.

Socio-emotional wellbeing was assessed in the Millennium Cohort Study by the Strengths and Difficulties Questionnaire (SDQ), a 25-item measure completed by the main respondent (usually the mother). These 25 questions comprise 5 scales of children’s behaviour (conduct problems, hyperactivity, emotional problems, peer problems, and prosocial behaviours), containing 5 questions each. We use the total difficulties score, which is the sum of four scales (peer problems, conduct disorders, hyperactivity, and emotional problems) and applied a validated cut-off to distinguish ‘normal’ from ‘borderline-abnormal’ scores. We also analysed pro-social scores at ages 7, 11 and 14, again using a standard cut-off to distinguish normal from borderline-abnormal scores since prosocial behaviours could be affected by school closures due to possible reduced socialisation [35].

### Socio-economic circumstances

Mother’s education is our primary measure of the cohort member’s socio-economic circumstances. We distinguish cohort members whose mothers had high (a university degree), moderate (A-levels, GCSE grades A-C, a diploma or equivalent) or low (GCSE grades D-G, equivalent or none) levels of qualifications. In sensitivity analyses, we examine differences by equivalized household income quintiles and by deciles of the Index of Multiple Deprivation, a neighbourhood deprivation measure.

### Confounders

We adjusted for a range of characteristics to account for differences between cohort members surveyed before or after the school holidays. We include sex as a binary variable, child’s age in years and completed months, a measure of ethnicity based on six categories (White, Mixed, Indian, Pakistani/Bangladeshi, Black/Black British and Other (including Chinese), and a categorical variable for the four UK countries (England, Wales, Scotland, and Northern Ireland).

### Analysis

First, we fitted separate regression models for each of the three Millennium Cohort Study sweeps with terms for mother’s education level and period (pre- or post-summer holiday). We included in these models all the variables that were fitted in the regressions used to derive our inverse probability weighting (i.e., gender, ethnicity, country), plus age expressed in years and completed months, to adjust for possible differences in sample composition between the two periods. We call these models 1a, 2a, 3a for Sweeps 4, 5 and 6 respectively. Next, we added an interaction term between mother’s education and period to identify whether inequalities in outcomes widened between the two periods examined. We call these models 1b, 2b and 3b.

For verbal cognitive ability scores the equation for 1b, 2b and 3b is the following:

1 $${Sab}_i={a}_o+{b}_1{M}_i+{b}_2{ME}_i+{b}_3\left({M}_i\ast {ME}_i\right)+{b}_4{G}_i+{b}_5{A}_i+{b}_6{E}_i+{b}_7{C}_i+{e}_i$$

where for a cohort member l i: *a*_*o*_ shows the intercept, *M* is a binary variable where 0 represents the pre summer holidays period and 1 the post summer period, *ME* is a categorical variable representing mother’s education split into three levels as described above while M*ME denotes the interaction between mother’s education and the binary variable on pre-and post-holiday periods. *G* is a binary variable for sex, *A* represents cohort members’ age in years and completed months, *E* and C are categorical variables for ethnicity and country respectively and *e* represents the error term. We use age-standardised scores but, in line with standard practice [36], we also adjust for cohort members’ age so that we can directly compare scores collected before and after the summer holidays. Scores are standardised using three-month age bands; including cohort member’s age in years and completed months as covariate controls for any variation within the bands. We used non-standardised ability scores in a sensitivity analysis.

For mental health, we followed a similar specification in terms of the exposure and other predictor variables, but since SDQ is represented by a binary variable we fitted a logistic regression model for each Millennium Cohort Study sweep:

2 $${SDQ}_i={a}_o+\exp \left({b}_1{M}_i\right)+\exp \left({b}_2{ME}_i\right)+\exp \left({b}_3\left({Z}_M\ast {Z}_{ME}\right)+\exp \left({b}_4{G}_i\right)+\exp \right({b}_5{A}_i+\exp \left({b}_6{E}_i\right)+\exp \left({b}_7{C}_i\right)+{e}_i$$

where Z_M_= exp(*b*_1_*M*_*i*_) and Z_MA_= exp(*b*_2_*ME*_*i*._)

We fitted models of the same form for SDQ prosocial scores (Additional file 1, Table A5c).

In all analyses we used survey weights to correct for the sample design in the current wave and non-response at the previous wave so that the results reflect the composition of the UK population at the relevant ages. We combined these with stabilised inverse probability weights to further account for differences by age, sex, country and socio-economic position in the composition of the samples in the pre- and post-holiday periods. Full details of the weighting strategy are provided in the Additional file 1. Separate weights were calculated for each outcome as the pattern of missingness and therefore the sample composition could differ between outcomes.

## Results

Table 1 shows the numbers of observations in the samples used in the regression analysis, broken down by period (pre- and post-summer holidays), UK country, survey Sweep and outcome variable. Further details of the weighting strategy and sample size broken down by calendar month are provided in the Additional file 1 Table A1a and b. Descriptive statistics for the outcome measures in the whole sample at each wave and a comparison of the covariate distributions in the unweighted and weighted samples are provided in Additional file 1 Table A2. Relevant flow charts were also drawn (Table A3a,b,c).

**Table 1:** Pre- and post-holidays sample statistics for Word ability, SDQ, SDQ-prosocial) by country and child age/survey wave

		Word ability			SDQ			SDQ pro-social		
	Country	Pre summer holidays	Post summer holidays	Total	Pre summer holidays	Post summer holidays	Total	Pre summer holidays	Post summer holidays	Total
**Sweep 4 (Age 7)**	**England**	2084	410	**2494**	2017	390	**2407**	2040	395	**2435**
	**Wales**	451	173	**624**	487	207	**694**	488	208	**696**
	**Scotland**	321	460	**781**	321	452	**773**	322	456	**778**
	**N. Ireland**	434	359	**793**	435	361	**796**	436	364	**800**
	**Total**	**3290**	**1402**	**4692**	**3260**	**1410**	**4670**	**3286**	**1486**	**4709**
**Sweep 5 (Age 11)**	**England**	1304	260	**1564**	1255	246	**1501**	1261	249	**1510**
	**Wales**	448	110	**558**	443	108	**551**	445	108	**553**
	**Scotland**	229	381	**610**	233	380	**613**	233	380	**613**
	**N. Ireland**	198	408	**606**	199	408	**607**	199	410	**609**
	**Total**	**2179**	**1159**	**3338**	**2130**	**1142**	**3272**	**2138**	**1147**	**3285**
**Sweep 6 (Age 14)**	**England**	1531	640	**2171**	1701	642	**2343**	1702	644	**2346**
	**Wales**	268	196	**464**	281	193	**474**	281	193	**474**
	**Scotland**	257	200	**457**	270	203	**473**	270	203	**473**
	**N. Ireland**	169	237	**406**	163	247	**410**	163	247	**410**
	**Total**	**2225**	**1273**	**3498**	**2415**	**1285**	**3700**	**2416**	**1287**	**3703**

**Fig. 1:**
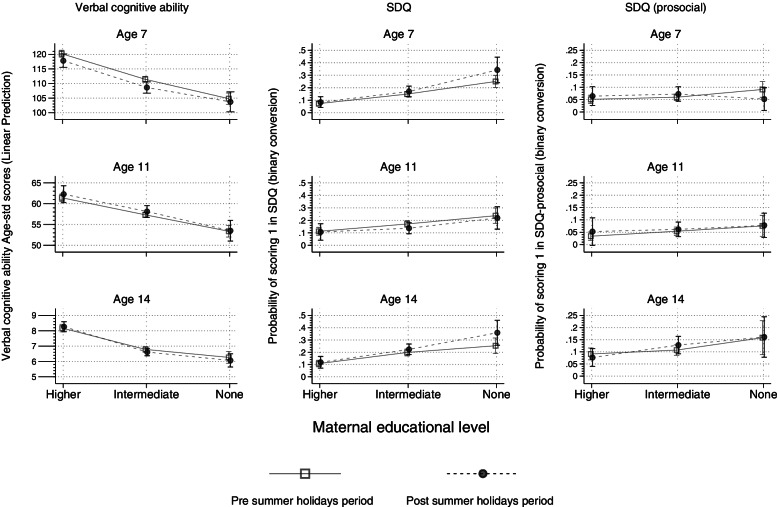
Inequalities in mental health and verbal cognitive ability (pre- and post- holidays) -Ages (7, 11, 14)

Our main findings in terms of odds ratios are presented in Table 2. Figure 1 depicts predictive margins using the margins command in Stata 17.0. Detailed results are presented in the Additional file 1, Table A5a, b, and c.

**Table 2:** Effects of mother’s education, pre/post school holidays) and their interaction on verbal cognitive ability and SDQ scores – (Ages 7, 11, 14)

		Effect estimates			Sample sizes
	Age	Mother’s Education^a^	School holidays^b^	Interaction^c^	
**SDQ**^**d**^	7	5.2 (3.3, 8.1)	1.3 (1.0, 1.6)	1.4 (0.6, 3.2)	4670
	11	2.5 (1.5, 3.9)	0.8 (0.6, 1.1)	0.9 (0.4, 2.6)	3372
	14	3.5 (2.3, 5.2)	1.3 (1.0, 1.6)	1.5 (0.7, 3.2)	3700
**SDQ-prosocial**^**d**^	7	1.2 (0.7,2.2)	1.1 (0.7,1.6)	0.4 (0.1, 1.5)	4709
	11	1.9 (0.8,4.2)	1.2 (0.7, 1.9)	0.7 (0.1,3.1)	3285
	14	2.1 (1.3,3.6)	1.1 (0.8, 1.5)	1.2 (0.5, 3.5)	3703
**Verbal cognitive ability**^**e**^	7	−14.7 (− 17.2, − 12.3)	− 2.4 (− 5.0, 0.2)	1.3 (− 3.3, 6.0)	4692
	11	−8.4 (− 10.2, − 6.6)	1.2 (−1.3, 3.9)	−0.7 (− 4.3, 2.8)	3398
	14	−2.1 (− 2.4, − 1.7)	0.1 (− 0.3, 0.5)	−0.3 (− 1.0, 0.4)	3498

^a^Comparison of lowest (GCSE grades D-G, equivalent or none) vs highest (university degree) level of mother’s education

^b^Comparison of children measured in the 2–3 months after vs. 2–3 months before the school summer holidays

^c^Interaction of lowest education and post-holiday period (Reference category: highest education category)

^d^Odds Ratio (95 CI)

^e^*b* (95 CI)

Table 2 presents the effect estimates for mother’s education (column 1) and the school summer holidays (column 2) on verbal cognitive ability and SDQ scores, adjusted for sex, ethnic and national differences in the composition of the pre- and post-holiday samples. The final column presents the coefficients of the interaction terms between school holidays and maternal education. The odds of borderline/abnormal total and prosocial SDQ scores were higher for cohort members with less educated mothers at each age. The odds of having a borderline/abnormal total difficulties score were higher among those surveyed after vs. before the school holidays at ages 7 and 14, but not at age 11. There were no substantial differences in pro-social behaviours among those surveyed before/after the school holidays. Verbal cognitive ability is lower for cohort members with less educated mothers at age 7, 11 and 14. At age 7, verbal cognitive ability scores were lower among those measured after the school holidays compared to those measured before. This was not the case at ages 11 or 14. Overall, the effects of maternal education on verbal cognitive ability and SDQ scores are greater than the effects of school summer holidays.

Figure 1 presents the socio-economic gradient (according to mother’s academic qualifications, X-axis) in verbal cognitive ability scores and risks of poor socio-emotional wellbeing (Y-axis), stratified by those surveyed before (solid line) and after (dashed line) the summer holidays.

As can be seen in Fig. 1 and Table 2 (Interaction column), the socio-economic gradient in total SDQ difficulties appeared to widen over the school holidays ages 7 [OR:1.4; 95% CI(0.6, 3.2)] and 14 [1.5(0.7, 3.2)], but the confidence intervals from the interaction term were wide. Inequalities in prosocial difficulties appear to narrow at age 7 [0.4(0.1, 1.5)] and slightly widen at age 14 [1.2(0.5, 3.5)] but again with wide confidence intervals. The socio-economic gradient in verbal cognitive ability does not widen over the school holidays. The negative coefficients from the interaction terms in Table 2 indicate a slight narrowing of inequalities at ages 11 [b:-0.7; 95%CI(− 4.3, 2.8)] and 14 [− 0.3 (− 10.4, 0.4)], but the changes were very small and the confidence intervals wide. Sensitivity analyses using non age standardised ability scores, (Additional file 1, Table A4) and income and neighbourhood deprivation in place of mother’s education (Additional file 1, Tables A6–7) found similar results.

## Discussion

This Millennium Cohort Study analysis is the first analysis of UK-wide data on the impacts of school summer holiday closures on inequalities in children and young people’s mental health and verbal cognitive ability. We observed marked inequalities in children and young people’s mental health and verbal cognitive ability according to maternal education and other measures of social position, but the evidence is mixed, with inconsistent results across different age groups. The increase in population-level SDQ total difficulties scores over the summer holidays was greater among disadvantaged groups, leading to a widening of inequality at ages 7 and 14, but not at age 11. Analysis suggests a relative decline in prosocial behaviour at age 14, while there was a relative improvement at age 7 and 11. We found younger children’s verbal cognitive ability declined by a small amount over the holidays regardless of socio-economic background. No decline was found in verbal cognitive ability over the holidays for other older age groups. We found no evidence that inequalities in verbal cognitive ability widened over the school summer holidays. As is often the case with interaction analyses, statistical power was limited, so the differences in the changes in mental health and verbal cognitive ability are imprecisely measured, and we cannot be confident that they reflect a real narrowing or widening of inequality.

These findings are consistent with the international evidence that has accumulated since the last systematic review was published in 1996. Several recent analyses of large USA datasets examining differences in cognitive ability after school summer closures between socio-economic groups have found different direction of results across school years [5–7]. Recent studies have questioned some of the methods used in earlier research, emphasising that measuring inequalities in attainment is problematic, and that heterogeneity in reported findings reflects the diverse approaches taken to measurement and analysis [3, 4, 19]. A strength of our study is that this secondary analysis uses high quality nationally representative survey data, covering socio-emotional wellbeing as well as cognitive ability, among a cohort of children and young people who were measured at three separate ages covering both primary and secondary school-age. Importantly, we were able to compare pre- and post-holiday scores on the same measures within each age group, so our results should not be subject to some of the scaling problems that have affected earlier studies of summer learning loss [3, 4, 19].

A limitation of our study is that using the Millennium Cohort Study data from a restricted set of pre- and post-holiday months risks incorporating bias and we were unable to measure changes within cohort members. However, the rich socio-economic and demographic data and the sampling and attrition weights available within the Millennium Cohort Study allowed a weighting strategy which we believe has adequately compensated for this. As we only know the month in which each interview took place, rather than the exact day, some interviews at the end of each pre-holiday period and some at the end of the school holiday will be misclassified. Although we were able to compare pre- and post-holiday scores on the same measures within each age group, there remains a risk of under-estimating effects on inequalities due to floor or ceiling effects. The power of the study to detect small effects is constrained by the sample size, and with the numbers available we were unable to conduct analyses stratified according to levels of ability. Although our measures of cognitive ability were based on reading tests, the mental health measures were based on reports by parents. It is possible that reporting bias may have contributed to differences in scores given by those interviewed before and after the summer holidays. Moreover, SDQ ratings are based on the child’s behaviour over the previous 6 months so this might have biased our results; however, it is more likely parents would be replying based on the most recent behaviour of their child. We use outcome data collected between 2009 and 2016, so the results may not generalise to later periods if the experiences of children during school holidays have changed – for example, as a result of widening socio-economic inequalities or a rise in child poverty.

School closures were among the most widespread measures taken to control the spread of infection in the first wave of the COVID-19 pandemic, affecting 1.5 billion children in 190 countries in April 2020 [37], despite a lack of evidence from previous research [38], or support from modelling studies [39], to suggest that closing schools has a large effect on infection rates. Surveys conducted during the UK restrictions revealed wide differences in the nature and level of support for learning available to children from different backgrounds (for example, those attending state and private schools), and corresponding disparities in the ability of children from more or less affluent families to make use of the support provided [40]. Concerns over the impacts school closures on mental health, due to the reduction in pastoral care, and structure and routine, have contributed to calls for schools to prioritise mental wellbeing upon their return to school [41]. A shift in approach towards keeping schools open for as long as possible, and prioritising their reopening during the easing of restrictions, was evident during the second wave of the pandemic in the UK in Autumn and Winter 2020.

Our findings from the UK are consistent with other research evidence that suggests that adverse impacts do not automatically follow from the cessation of schooling over the summer. A large longitudinal study in England reported little change in pupil wellbeing across the first period of COVID-related school closures in Spring 2020 [42]. However, negative effects of school closures are being reported as further research emerges. For example, a USA study found pandemic related negative impacts on young people’s mental health [43], and learning and behaviour problems were found in a study of Italian school children [44]. These studies and a number of other surveys [45] conducted during the pandemic have reported widely differing experiences of schooling during the period when schools were closed, which may mean that pandemic related closures have a larger effect on inequalities in learning than we observe following routine summer closures when most children have a break from formal education. Evidence has also begun to emerge of attainment losses as a result of the lengthy closures in Spring and Summer 2020 [46, 47]. Future research should focus on establishing whether those early indications of adverse effects are real, what they mean for inequalities in health and educational attainment, and what are the mechanisms that cause them, so that interventions can be appropriately designed and targeted, in order to mitigate the adverse effects of future extended closures.

## Conclusions

The results of our analyses of UK children are consistent with the mixed picture emerging from the international literature. They suggest that in normal circumstances, school summer holidays do not lead to significant additional educational disadvantage. School closures during the COVID19 pandemic may have a larger effect, because they last longer, because inequalities in support for learning have been more pronounced during the pandemic-related closures than they are during summer holidays in general, or because disadvantaged children tend to live in households more severely affected by the social and economic disruption of the pandemic. The possibility of such effects should be an important focus of future research and monitoring.

## Supplementary Information

**Additional file 1.**

## Data Availability

The data used in this work is held by the UK Data Service (10.5255/UKDA-SN-4683-1; 10.5255/UKDA-SN-6411-6; 10.5255/UKDA-SN-7464-2; 10.5255/UKDA-SN-8156-2). They can be accessed upon registration with UK-data-Archive (https://www.data-archive.ac.uk/).
