# Supplementary material for: The effect of school summer holidays on inequalities in children and young people’s mental health and cognitive ability in the UK using data from the millennium cohort study
Source: BMC Public Health. 2022 Jan 22;22:154. doi: 10.1186/s12889-022-12540-2 (PMC8782677; doi:10.1186/s12889-022-12540-2)
Supplement: Supplementary file 1 — Additional file 1. [file 12889_2022_12540_MOESM1_ESM.docx]

**Supporting Information (S2 File)**

Table A.1a Sample sizes for MCS analyses: overall and for pre- and post-holiday periods, for each survey wave and UK country

|  | **Country** | **Jan** | **Feb** | **March** | **April** | **May** | **June** | **July** | **Aug** | **Sept** | **Oct** | **Nov** | **Dec** | **Total pre** | **Total post** | **Total(pre-post)*** | **Total Sample** |
| --- | --- | --- | --- | --- | --- | --- | --- | --- | --- | --- | --- | --- | --- | --- | --- | --- | --- |
| **Sweep 4 (Age 7)** | **England** | 20 | 589 | 1,230 | 1,803 | 1,675 | 1,292 | 954 | 746 | 363 | 91 | 7 | 8 | **2,246** | **454** | **2,700** | **8,778** |
|  | **Perc row** | 0.23 | 6.71 | 14.01 | 20.54 | 19.08 | 14.72 | 10.87 | 8.50 | 4.14 | 1.04 | 0.08 | 0.09 | **25.59** | **5.17** | **30.76** | **100.00** |
|  | **Perc column** | 28.17 | 84.63 | 86.56 | 73.14 | 71.58 | 71.07 | 74.65 | 66.37 | 33.86 | 12.69 | 1.39 | 4.55 | **63.82** | **30.31** | **53.82** | **64.16** |
|  | **Wales** | 0 | 104 | 191 | 328 | 341 | 238 | 259 | 273 | 157 | 53 | 7 | 0 | **497** | **210** | **707** | **1,951** |
|  | **Perc row** | 0.00 | 5.33 | 9.79 | 16.81 | 17.48 | 12.20 | 13.28 | 13.99 | 8.05 | 2.72 | 0.36 | 0.00 | **25.47** | **10.76** | **36.24** | **100.00** |
|  | **Perc column** | 0.00 | 14.94 | 13.44 | 13.31 | 14.57 | 13.09 | 20.27 | 24.29 | 14.65 | 7.39 | 1.39 | 0.00 | **14.12** | **14.02** | **14.09** | **14.26** |
|  | **Scotland** | 51 | 3 | 0 | 164 | 133 | 31 | 15 | 78 | 385 | 369 | 241 | 128 | **328** | **463** | **791** | **1,598** |
|  | **Perc row** | 3.19 | 0.19 | 0.00 | 10.26 | 8.32 | 1.94 | 0.94 | 4.88 | 24.09 | 23.09 | 15.08 | 8.01 | **20.53** | **28.97** | **49.50** | **100.00** |
|  | **Perc column** | 71.83 | 0.43 | 0.00 | 6.65 | 5.68 | 1.71 | 1.17 | 6.94 | 35.91 | 51.46 | 47.91 | 72.73 | **9.32** | **30.91** | **15.77** | **11.68** |
|  | **N. Ireland** | 0 | 0 | 0 | 170 | 191 | 257 | 50 | 27 | 167 | 204 | 248 | 40 | **448** | **371** | **819** | **1,354** |
|  | **Perc row** | 0.00 | 0.00 | 0.00 | 12.56 | 14.11 | 18.98 | 3.69 | 1.99 | 12.33 | 15.07 | 18.32 | 2.95 | **33.09** | **27.40** | **60.49** | **100.00** |
|  | **Perc column** | 0.00 | 0.00 | 0.00 | 6.90 | 8.16 | 14.14 | 3.91 | 2.40 | 15.58 | 28.45 | 49.30 | 22.73 | **12.73** | **24.77** | **16.32** | **9.90** |
|  | **Total** | **71** | **696** | **1,421** | **2,465** | **2,340** | **1,818** | **1,278** | **1,124** | **1,072** | **717** | **503** | **176** | **3,519** | **1,498** | **5,017** | **13,681** |
| **Sweep 5 (Age 11)** | **England** | 15 | 2029 | 1,421 | 1,768 | 1,162 | 839 | 570 | 388 | 172 | 120 | 49 | 29 | **1,409** | **292** | **1,701** | **8,562** |
|  | **Perc row** | 0.18 | 23.70 | 16.60 | 20.65 | 13.57 | 9.80 | 6.66 | 4.53 | 2.01 | 1.40 | 0.57 | 0.34 | **16.46** | **3.41** | **19.87** | **100.00** |
|  | **Perc column** | 16.48 | 82.65 | 73.29 | 78.61 | 81.20 | 71.71 | 65.29 | 56.56 | 22.54 | 14.71 | 10.29 | 17.79 | **66.31** | **24.25** | **51.10** | **65.30** |
|  | **Wales** | 14 | 250 | 218 | 322 | 168 | 223 | 233 | 214 | 62 | 51 | 37 | 24 | **456** | **113** | **569** | **1,816** |
|  | **Perc row** | 0.77 | 13.77 | 12.00 | 17.73 | 9.25 | 12.28 | 12.83 | 11.78 | 3.41 | 2.81 | 2.04 | 1.32 | **25.11** | **6.22** | **31.33** | **100.00** |
|  | **Perc column** | 15.38 | 10.18 | 11.24 | 14.32 | 11.74 | 19.06 | 26.69 | 31.20 | 8.13 | 6.25 | 7.77 | 14.72 | **21.46** | **9.39** | **17.09** | **13.85** |
|  | **Scotland** | 41 | 51 | 178 | 51 | 7 | 0 | 5 | 55 | 331 | 430 | 225 | 75 | **58** | **386** | **444** | **1,449** |
|  | **Perc row** | 2.83 | 3.52 | 12.28 | 3.52 | 0.48 | 0.00 | 0.35 | 3.80 | 22.84 | 29.68 | 15.53 | 5.18 | **4.00** | **26.64** | **30.64** | **100.00** |
|  | **Perc column** | 45.05 | 2.08 | 9.18 | 2.27 | 0.49 | 0.00 | 0.57 | 8.02 | 43.38 | 52.70 | 47.27 | 46.01 | **2.73** | **32.06** | **13.34** | **11.05** |
|  | **N. Ireland** | 21 | 125 | 122 | 108 | 94 | 108 | 65 | 29 | 198 | 215 | 165 | 35 | **202** | **413** | **615** | **1,285** |
|  | **Perc row** | 1.63 | 9.73 | 9.49 | 8.40 | 7.32 | 8.40 | 5.06 | 2.26 | 15.41 | 16.73 | 12.84 | 2.72 | **15.72** | **32.14** | **47.86** | **100.00** |
|  | **Perc column** | 23.08 | 5.09 | 6.29 | 4.80 | 6.57 | 9.23 | 7.45 | 4.23 | 25.95 | 26.35 | 34.66 | 21.47 | **9.51** | **34.30** | **18.47** | **9.80** |
|  | **Total** | **91** | **2455** | **1,939** | **2,249** | **1,431** | **1,170** | **873** | **686** | **763** | **816** | **476** | **163** | **2,125** | **1,204** | **3,329** | **13,112** |
| **Sweep 6 (Age 14)** | **England** | 122 | 735 | 855 | 1,162 | 1,090 | 874 | 1,008 | 760 | 436 | 317 | 216 | 57 | **1,882** | **753** | **2,635** | **7,632** |
|  | **Perc row** | 1.60 | 9.63 | 11.20 | 15.23 | 14.28 | 11.45 | 13.21 | 9.96 | 5.71 | 4.15 | 2.83 | 0.75 | **24.66** | **9.87** | **34.53** | **100.00** |
|  | **Perc column** | 40.40 | 65.33 | 68.79 | 71.20 | 76.54 | 76.60 | 83.03 | 73.86 | 57.67 | 40.64 | 32.38 | 22.71 | **74.45** | **52.77** | **66.62** | **58.21** |
|  | **Wales** | 13 | 169 | 166 | 260 | 196 | 158 | 141 | 189 | 122 | 93 | 51 | 41 | **299** | **215** | **514** | **1,599** |
|  | **Perc row** | 0.81 | 10.57 | 10.38 | 16.26 | 12.26 | 9.88 | 8.82 | 11.82 | 7.63 | 5.82 | 3.19 | 2.56 | **18.70** | **13.45** | **32.15** | **100.00** |
|  | **Perc column** | 4.30 | 15.02 | 13.35 | 15.93 | 13.76 | 13.85 | 11.61 | 18.37 | 16.14 | 11.92 | 7.65 | 16.33 | **11.83** | **15.07** | **13.00** | **12.19** |
|  | **Scotland** | 106 | 120 | 107 | 100 | 46 | 28 | 15 | 45 | 163 | 154 | 245 | 107 | **174** | **208** | **382** | **1,236** |
|  | **Perc row** | 8.58 | 9.71 | 8.66 | 8.09 | 3.72 | 2.27 | 1.21 | 3.64 | 13.19 | 12.46 | 19.82 | 8.66 | **14.08** | **16.83** | **30.91** | **100.00** |
|  | **Perc column** | 35.10 | 10.67 | 8.61 | 6.13 | 3.23 | 2.45 | 1.24 | 4.37 | 21.56 | 19.74 | 36.73 | 42.63 | **6.88** | **14.58** | **9.66** | **9.43** |
|  | **N. Ireland** | 61 | 101 | 115 | 110 | 92 | 81 | 50 | 35 | 35 | 216 | 155 | 46 | **173** | **251** | **424** | **1,097** |
|  | **Perc row** | 5.56 | 9.21 | 10.48 | 10.03 | 8.39 | 7.38 | 4.56 | 3.19 | 3.19 | 19.69 | 14.13 | 4.19 | **15.77** | **22.88** | **38.65** | **100.00** |
|  | **Perc column** | 20.20 | 8.98 | 9.25 | 6.74 | 6.46 | 7.10 | 4.12 | 3.40 | 4.63 | 27.69 | 23.24 | 18.33 | **6.84** | **17.59** | **10.72** | **8.37** |
|  | **Total** | **302** | **1125** | **1,243** | **1,632** | **1,424** | **1,141** | **1,214** | **1,029** | **756** | **780** | **667** | **251** | **2,528** | **1,427** | **3,955** | **11,564** |

Note:* This number shows the total number of observations that correspond to the representative months for pre- and post- summer holiday period by UK country. However, it does not represent the final number used in the models for reasons explained in the “Weighting strategy” section.

Table A.1b Sample sizes by outcome variable and sweep

| **Outcome/Sweep** | **Sweep 4** | **Sweep 5** | **Sweep 6** |
| --- | --- | --- | --- |
| **Word ability** | 4,692 | 3,338 | 3,498 |
| **SDQ** | 4,670 | 3,372 | 3,700 |
| **SDQ prosocial** | 4,709 | 3,285 | 3,703 |

Table A.2 Covariate balance checks

|  |  | **Sweep 4 (Age 7)** | | | | | | | | **Sweep 5 (Age 11)** | | | | | | | | **Sweep 6 (Age 14)** | | | | | | | |
| --- | --- | --- | --- | --- | --- | --- | --- | --- | --- | --- | --- | --- | --- | --- | --- | --- | --- | --- | --- | --- | --- | --- | --- | --- | --- |
|  |  | **Unweighted Sample** | | | | | **Weighted** | | | **Unweighted Sample** | | | | | **Weighted** | | | **Unweighted Sample** | | | | | **Weighted** | | |
|  |  | **Count** | | | **Percentage (%)** | | **Count** | **Percentage (%)** | | **Count** | | | **Percentage (%)** | | **Count** | **Percentage (%)** | | **Count** | | | **Percentage (%)** | | **Count** | **Percentage (%)** | |
| **Factor** | **Value** | **Pre** | **Post** | **Total** | **Pre** | **Post** | **Total** | **Pre** | **Post** | **Pre** | **Post** | **Total** | **Pre** | **Post** | **Total** | **Pre** | **Post** | **Pre** | **Post** | **Total** | **Pre** | **Post** | **Total** | **Pre** | **Post** |
| **Sample** |  | **3,519** | **1,498** | **5,017** | **29.9** | **70.1** | **4,848** | **50.0** | **50.0** | **2,303** | **1,204** | **3,507** | **65.7** | **34.3** | **3,398** | **50.8** | **49.2** | **2,635** | **1,427** | **4,062** | **64.9** | **35.1** | **3,896** | **50.0** | **50.0** |
| **Outcome** |  |  |  |  | **Mean score** | |  | **Mean score** | |  |  |  | **Mean score** | |  | **Mean score** | |  |  |  | **Mean score** | |  | **Mean score** | |
| **Word ability (age-standardised)** | | **3,416** | **1,438** | **4,854** | **112.5** | **108.3** | **4,692** | **112.6** | **109.8** | **2,258** | **1,184** | **3,442** | **58.0** | **59.3** | **3,338** | **57.6** | **57.6** | **2,328** | **1,316** | **3,644** | **7.2** | **7.0** | **3,498** | **7.0** | **6.9** |
|  |  |  |  |  | **Proportion** | |  | **Proportion** | |  |  |  | **Proportion** | |  | **Proportion** | |  |  |  | **Proportion** | |  | **Proportion** | |
| **SDQ** |  | **3,383** | **1,446** | **4,829** | **14.5** | **13.0** | **4,673** | **14.7** | **17.9** | **2,206** | **1,166** | **3,372** | **16.1** | **13.7** | **3,272** | **17.2** | **14.7** | **2,527** | **1,332** | **3,859** | **17.3** | **18.5** | **3,700** | **19.0** | **22.2** |
| **SDQ-prosocial** |  | **3,410** | **1,459** | **4,869** | **0.06** | **0.06** | **4,712** | **0.06** | **0.07** | **2,214** | **1,172** | **3,386** | **0.05** | **0.05** | **3,285** | **0.05** | **0.06** | **2,528** | **1,334** | **3,862** | **0.10** | **0.10** | **3,703** | **0.11** | **0.13** |
|  |  | **Count** | | | **Percentage (%)** | | **Count** | **Percentage (%)** | | **Count** | | | **Percentage (%)** | | **Count** | **Percentage (%)** | | **Count** | | | **Percentage (%)** | | **Count** | **Percentage (%)** | |
| **Modifier** |  | **Pre** | **Post** | **Total** | **Pre** | **Post** | **Total** | **Pre** | **Post** | **Pre** | **Post** | **Total** | **Pre** | **Post** | **Total** | **Pre** | **Post** | **Pre** | **Post** | **Total** | **Pre** | **Post** | **Total** | **Pre** | **Post** |
| **Mother`s education** | **Higher** | 882 | 454 | **1,336** | 66.0 | 34.0 |  | 50.3 | 49.7 | 600 | 338 | **938** | 64.0 | 36.0 |  | 53.7 | 46.3 | 797 | 396 | **1,193** | 66.8 | 33.2 |  | 50.3 | 49.7 |
|  | **Intermediate** | 1,988 | 795 | **2,783** | 71.4 | 28.6 |  | 50.3 | 49.7 | 1,273 | 636 | **1,909** | 66.7 | 33.3 |  | 50,4 | 49.6 | 1,432 | 789 | **2,221** | 64.5 | 35.3 |  | 50.1 | 49.9 |
|  | **None** | 631 | 242 | **873** | 72.3 | 27.7 |  | 48.7 | 51.2 | 420 | 222 | **642** | 65.4 | 34.6 |  | 49.2 | 50.8 | 392 | 228 | **620** | 63.2 | 36.8 |  | 49.7 | 50.3 |
|  | **Total** | 3,501 | 1,491 | **4,992** | 70.1 | 29.9 | **4,848** | 50.0 | 50.0 | 2,293 | 1,196 | **3,489** | 65.7 | 34.3 | **3,398** | 50.8 | 49.2 | 2,621 | 1,413 | **4,034** | 65.0 | 35.0 | **3,896** | 50.0 | 50.0 |
| **Covariates** |  | **Pre** | **Post** | **Total** | **Pre** | **Post** | **Total** | **Pre** | **Post** | **Pre** | **Post** | **Total** | **Pre** | **Post** | **Total** | **Pre** | **Post** | **Pre** | **Post** | **Total** | **Pre** | **Post** | **Total** | **Pre** | **Post** |
| **Age** |  | 3,514 | 1,495 | **5,009** | 7.2 | 7.2 | **4,841** | 7.2 | 7.3 | 2,303 | 1,204 | **3,507** | 11.3 | 11.2 | **3,398** | 11.3 | 11.5 | 2,635 | 1,427 | **4,062** | 14.3 | 14.4 |  | 14.3 | 14.5 |
| **Sex** | **Male** | 1,675 | 740 | **2,415** | 69.4 | 30,64 |  | 50.0 | 50.0 | 1,140 | 590 | **1,730** | 65.9 | 34.1 |  | 51.3 | 48.7 | 1,233 | 709 | **1,942** | 63.5 | 36.5 |  | 50.1 | 49.9 |
|  | **Female** | 1,725 | 729 | **2,454** | 70.3 | 29.7 |  | 50.0 | 50.0 | 1,190 | 592 | **2,374** | 64.8 | 35.2 |  | 50.3 | 49.7 | 1,295 | 676 | **1,971** | 65.7 | 34.3 |  | 49.6 | 50.4 |
|  | **Total** | 3,400 | 1,469 | **4,869** | 69.8 | 30.2 | **4,848** | 50.0 | 50.0 | 2,230 | 1,182 | **4,594** | 65.4 | 34.6 | **3,398** | 50.8 | 49.2 | 2,528 | 1,385 | **3,913** | 64.6 | 35.4 | **3,896** | 49.9 | 50.1 |
| **Ethnicity** | **White** | 2,829 | 1,313 | **4,142** | 68.3 | 31.7 |  | 50.0 | 50.0 | 1,869 | 1,060 | **2,929** | 63.8 | 36.2 |  | 51.1 | 48.9 | 2,141 | 1,171 | **3,312** | 64.6 | 35.4 |  | 50.1 | 49.9 |
|  | **Mixed** | 176 | 37 | **213** | 82.6 | 17.4 |  | 50.1 | 49.9 | 94 | 41 | **135** | 69.6 | 30.4 |  | 50.2 | 49.8 | 125 | 65 | **190** | 65.8 | 34.2 |  | 49.6 | 50.3 |
|  | **Indian** | 84 | 34 | **118** | 71.2 | 28.8 |  | 49.3 | 50.6 | 51 | 12 | **63** | 81.0 | 19.1 |  | 48.4 | 51.6 | 62 | 36 | **98** | 63.3 | 36.7 |  | 49.9 | 50.1 |
|  | **P/Ban*** | 224 | 43 | **267** | 83.9 | 16.1 |  | 50.1 | 49.9 | 191 | 45 | **236** | 80.9 | 19.1 |  | 48.5 | 51.5 | 168 | 77 | **245** | 68.6 | 31.4 |  | 49.9 | 50.1 |
|  | **Black/BB**** | 131 | 41 | **172** | 76.2 | 23.8 |  | 50.0 | 50.0 | 50 | 27 | **77** | 64.9 | 35.1 |  | 49.7 | 50.3 | 77 | 47 | **124** | 62.1 | 37.9 |  | 49.7 | 50.3 |
|  | **Other***** | 75 | 30 | **105** | 71.4 | 28.6 |  | 50.5 | 49.5 | 48 | 18 | **66** | 72.7 | 27.3 |  | 51.2 | 48.8 | 62 | 31 | **93** | 66.7 | 33.3 |  | 49.2 | 50.8 |
|  | **Total** | 3,519 | 1,498 | **5,017** | 70.1 | 29.9 | **4,848** | 50.0 | 50.0 | 2,303 | 1,203 | **3,506** | 65.7 | 34.3 | **3,398** | 50.8 | 49.2 | 2,635 | 1,427 | **4,062** | 64.9 | 35.1 | **3,896** | 50.0 | 50.0 |
| **UK Country** | **England** | 2,246 | 454 | **2,700** | 83,19 | 16,81 |  | 50.0 | 50.0 | 1,409 | 292 | **1,701** | 82.8 | 17.2 |  | 50.9 | 49.1 | 1,882 | 753 | **2,635** | 71.4 | 28.6 |  | 50.3 | 49.7 |
|  | **Wales** | 497 | 210 | **707** | 70.3 | 29.7 |  | 50.1 | 49.9 | 456 | 113 | **569** | 80.1 | 19.9 |  | 49.8 | 50.2 | 299 | 215 | **514** | 58.2 | 41.8 |  | 50.1 | 49.9 |
|  | **Scotland** | 328 | 463 | **791** | 41.5 | 58.5 |  | 50.0 | 50.0 | 236 | 386 | **622** | 37.9 | 62.1 |  | 50.9 | 49.1 | 281 | 208 | **489** | 57.5 | 42.5 |  | 50.0 | 50.0 |
|  | **Ireland** | 448 | 371 | **819** | 54.7 | 45.3 |  | 50.0 | 50.0 | 202 | 413 | **615** | 32.9 | 67.2 |  | 50.9 | 49.1 | 173 | 251 | **424** | 40.8 | 59.2 |  | 50.3 | 49.7 |
|  | **Total** | 3,519 | 1,498 | **5,017** | 70.1 | 29.9 | **4,848** | 50.0 | 50.0 | 2,303 | 1,204 | **3,507** | 65.7 | 34.3 | **3,398** | 50.8 | 49.2 | 2,635 | 1,427 | **4,062** | 64.9 | 35.1 | **3,896** | 50.0 | 50.0 |

Note: Light greyed columns show percentages from the original and dark greyed from the balanced dataset we finally used in our analysis after calculating IPW for that purpose. From Table 2 we can see that all key variables were seriously unbalanced. However, the IPW-adjusted sample, balances the data between pre- and post-summer holiday periods. The first row shows figures for our restricted sample comparing unweighted and weighted averages. The next three rows show the mean for our outcome variables pre- and post-summer holidays for the unweighted and weighted restricted sample. The rest of the rows show all covariates used in our analysis. The variables on children’s age by month, performs slightly worse when it is weighted. This is because it has not been used in weighting.

*Pakistani/Bangladeshi, **: Black/Black British. ***: Other (incl. Chinese)

**Table A.3a: Flow Chart by outcome (Sweep 4-Age 7)**

| **Word Ability**  MCS observations of cohort members (CMs) at baseline **(n=13,681)**  CMs outside SHPs **(n=8,028)**  CMs with missing data in main exposure **(n=60)**  CMs with complete data in main exposure **(n=13,588)**  CMs with complete data in all covariates and valid MCS weights **(n=12,720)**  CMs with missing data in covariates and non-valid weights **(n=868)**  CMs with complete data in all covariates and pre and post summer holidays periods (SHPs) and valid MCS and IPW weights  **(n=4,692; Pre SHP=3,290 Post SHP=1,402)** |
| --- |
| **SDQ**  MCS observations of cohort members (CMs) at baseline **(n=13,681)**  CMs outside SHPs **(n=8,010)**  CMs with missing data in main exposure **(n=60)**  CMs with complete data in main exposure **(n=13,588)**  CMs with complete data in all covariates and valid MCS weights **(n=12,680)**  CMs with missing data in covariates and non-valid weights **(n=908)**  CMs with complete data in all covariates and pre and post summer holidays periods (SHPs) and valid MCS and IPW weights  **(n=4,670; Pre SHP=3,260 Post SHP=1,410)** |
| **SDQ-Prosocial**  MCS observations of cohort members (CMs) at baseline **(n=13,681)**  CMs with missing data in main exposure **(n=60)**  CMs outside SHPs **(n=8,079)**  CMs with complete data in all covariates and valid MCS weights **(n=12,788)**  CMs with complete data in all covariates and pre and post summer holidays periods (SHPs) and valid MCS and IPW weights  **(n=4,709; Pre SHP=3,286 Post SHP=1,423)**  CMs with missing data in covariates and non-valid weights **(n=800)**  CMs with complete data in main exposure **(n=13,588)** |

**Table A.3b: Flow Chart by outcome (Sweep 5-Age 11)**

| **Word Ability**  MCS observations of cohort members (CMs) at baseline **(n=13,112)**  CMs outside SHPs **(n=8,989)**  CMs with missing data in main exposure **(n=82)**  CMs with complete data in main exposure **(n=13,030)**  CMs with complete data in all covariates and valid MCS weights **(n=12,327)**  CMs with missing data in covariates and non-valid weights **(n=703)**  CMs with complete data in all covariates and pre and post summer holidays periods (SHPs) and valid MCS and IPW weights  **(n=3,338; Pre SHP=2,179 Post SHP=1,159)** |
| --- |
| **SDQ**  MCS observations of cohort members (CMs) at baseline **(n=13,112)**  CMs outside SHPs **(n=8,842)**  CMs with missing data in main exposure **(n=82)**  CMs with complete data in main exposure **(n=13,030)**  CMs with complete data in all covariates and valid MCS weights **(n=12,114)**  CMs with missing data in covariates and non-valid weights **(n=916)**  CMs with complete data in all covariates and pre and post summer holidays periods (SHPs) and valid MCS and IPW weights  **(n=3,272; Pre SHP=2,130 Post SHP=1,142)** |
| **SDQ-Prosocial**  MCS observations of cohort members (CMs) at baseline **(n=13,112)**  CMs with missing data in main exposure **(n=82)**  CMs outside SHPs **(n=8,877)**  CMs with complete data in all covariates and valid MCS weights **(n=12,162)**  CMs with complete data in all covariates and pre and post summer holidays periods (SHPs) and valid MCS and IPW weights  **(n=3,285; Pre SHP=2,138 Post SHP=1,147)**  CMs with missing data in covariates and non-valid weights **(n=868)**  CMs with complete data in main exposure **(n=13,030)** |

**Table A.3c: Flow Chart by outcome (Sweep 6-Age 14)**

| **Word Ability**  MCS observations of cohort members (CMs) at baseline **(n=11,564)**  CMs outside SHPs **(n=6,244)**  CMs with missing data in main exposure **(n=66)**  CMs with complete data in main exposure **(n=11,498)**  CMs with complete data in all covariates and valid MCS weights **(n=9,742)**  CMs with missing data in covariates and non-valid weights **(n=1,756)**  CMs with complete data in all covariates and pre and post summer holidays periods (SHPs) and valid MCS and IPW weights  **(n=3,498; Pre SHP=2,225 Post SHP=1,273)** |
| --- |
| **SDQ**  MCS observations of cohort members (CMs) at baseline **(n=11,564)**  CMs outside SHPs **(n=6,434)**  CMs with missing data in main exposure **(n=66)**  CMs with complete data in main exposure **(n=11,498)**  CMs with complete data in all covariates and valid MCS weights **(n=10,134)**  CMs with missing data in covariates and non-valid weights **(n=1,364)**  CMs with complete data in all covariates and pre and post summer holidays periods (SHPs) and valid MCS and IPW weights  **(n=3,700; Pre SHP=2,415 Post SHP=1,285)** |
| **SDQ-Prosocial**  MCS observations of cohort members (CMs) at baseline **(n=11,564)**  CMs outside SHPs **(n=6,446)**  CMs with missing data in main exposure **(n=66)**  CMs with complete data in all covariates and valid MCS weights **(n=10,149)**  CMs with complete data in all covariates and pre and post summer holidays periods (SHPs) and valid MCS and IPW weights  **(n=3,703; Pre SHP=2,416 Post SHP=1,287)**  CMs with missing data in covariates and non-valid weights **(n=1,349)**  CMs with complete data in main exposure **(n=11,498)** |

**Weighting strategy:**

We employed a logit model where the outcome variable is time period (before and after school summer holidays) and regressors include all the confounders used in our main models, apart from cohort’s age in months. We used the same procedure for all three sweeps. In the weighting regression equation, we included all measures of socio-economic circumstances simultaneously, in order to use the same weight in our main and sensitivity analyses. The inclusion of all measures of socioeconomic circumstances improves data balance considerably; however, this means that our models include only those with no-missing data in these variables. Similarly, those with missing data in our exposure variables were excluded from our analysis. Therefore, since we have restricted our analysis to cohort members surveyed in the two months (three in the case of Scotland) before and the two months after the summer holidays in each sweep, our working sample sizes differ depending on the outcome variable used each time (Table A2.1b)

In our outcome models we use an interaction of mother’s education with period. We replace mother’s education with income quintiles and/or Index of Multiple Deprivation for the purposes of sensitivity analysis. However, since the three measures do not provide identical information on socio-economic circumstances, we have included them in our weighting models to make our sample more inclusive and comparable between the two periods examined and improve the comparability of our main and sensitivity analyses.

In our logit model to calculate stabilised IPW we have also included the weights provided by MCS. These weights account for sample design and non-response. Our core analysis is a repeated cross-sectional but the MCS weights have a longitudinal element as well as they account for non-response one sweep before. So, the overall MCS weight, for example at sweep 5 is a product of the sampling weight at sweep 5 and non-response weight for the sweep 4. The weight we have finally used in our analysis is the product of the overall MCS weight with the stabilised IPW described above. More detailed information on MCS sampling methods can be found in Mostafa (2014).

Table A.3 Regressions of reading ability on education level, period, and education level by period for children aged 7 and 11: sensitivity analysis comparing standardised with unstandardised reading ability scores

|  | **(Core)** | | **(Sensitivity)** | | **(Core)** | | | **(Sensitivity)** | |
| --- | --- | --- | --- | --- | --- | --- | --- | --- | --- |
|  | **S4 CM Word Reading Standard Score** | | **S4 CM Word Reading Ability Score** | | **S5 DV Verbal Sims standard score** | | | **S5 DV Verbal Sims ability score** | |
|  | **b** | **ci95** | **b** | **ci95** | **b** | **ci95** | | **b** | **ci95** |
| **Summer holidays After (Ref: Before)** | -2.406 | -4.984, 0.171 | -4.050 | -8.329, 0.228 | 0.870 | -1.353, 3.093 | | -3.447 | -9.623, 2.730 |
| **Higher** |  |  |  |  |  |  | |  |  |
| **Intermediate** | -8.772 | -10.27, -7.274 | -14.65 | -17.14, -12.15 | -4.097 | -5.139, -3.056 | | -7.804 | -10.12, -5.486 |
| **Lower** | -15.41 | -17.69, -13.13 | -25.90 | -29.77, -22.03 | -8.039 | -9.694, -6.384 | | -17.72 | -21.90, -13.54 |
| **Interaction Term (Ref: Higher)** |  |  |  |  |  |  | |  |  |
| **Intermediate** | -0.416 | -3.753, 2.920 | -0.818 | -6.391, 4.755 | -0.0768 | -2.704, 2.551 | | 3.631 | -3.798, 11.06 |
| **Lower** | 1.312 | -3.336, 5.961 | 2.581 | -5.127, 10.29 | -0.737 | -4.254, 2.781 | | 6.291 | -2.389, 14.97 |
| **Constant** | 154.6 | 131.6, 177.6 | 17.84 | -21.34, 57.03 | 89.42 | 67.34, 111.5 | | 82.06 | 27.80, 136.3 |
| **Observations** | 4,692 | | 4,692 | | 3,338 | | 3,398 | | |
| **R2 (Adjusted)** | 0.106 | | 0.109 | | 0.081 | | 0.044 | | |

Table A.4 Regressions of reading ability, SDQ total difficulties and prosocial scores on education level, period, and education level by period for children aged 7 (model 1a-b), 11 (model 2a-b) and 14 (model 3a-b)

Table A4a: Word ability scores, IPW-adjusted.

|  | **(1a)** | | **(1b)** | | **(2a)** | | **(2b)** | | **(3a)** | | **(3b)** | |
| --- | --- | --- | --- | --- | --- | --- | --- | --- | --- | --- | --- | --- |
|  | **S4 CM Word Reading Standard Score**  **Interaction: No** | | **S4 CM Word Reading Standard Score**  **Interaction: Yes** | | **S5 DV Verbal Sims standard score**  **Interaction: No** | | **S5 DV Verbal Sims standard score**  **Interaction: Yes** | | **S6: Word activity score out of 20**  **Interaction: No** | | **S6: Word activity score out of 20**  **Interaction: Yes** | |
|  | b | ci95 | b | ci95 | b | ci95 | b | ci95 | b | ci95 | b | ci95 |
|  |  |  |  |  |  |  |  |  |  |  |  |  |
| **Summer holidays After (Ref: Before)** | -2.401 | -3.949, -0.854 | -2.406 | -4.984, 0.171 | 0.659 | -0.574, 1.892 | 1.273 | -1.339, 3.884 | -0.118 | -0.344, 0.108 | 0.089 | -0.320, 0.497 |
| **Mother’s Education (Ref: Higher)** |  |  |  |  |  |  |  |  |  |  |  |  |
| **Intermediate** | -8.975 | -10.62, -7.326 | -8.772 | -10.35, -7.360 | -4.124 | -5.364, -2.884 | -4.097 | -5.139, -3.056 | -1.521 | -1.771, -1.272 | -1.390 | -1.680, -1.099 |
| **Lower** | -14.72 | -17.15, -12.30 | -15.41 | -17.72, -13.18 | -8.401 | -10.18, -6.618 | -8.039 | -9.694, -6.384 | -2.055 | -2.413, -1.697 | -1.909 | -2.359, -1.459 |
| **Interaction Term**  **Ref: Higher** |  |  |  |  |  |  |  |  |  |  |  |  |
| **Intermediate** |  |  | -0.416 | -3.753, 2.920 |  |  | -0.077 | -2.704, 2.551 |  |  | -0.262 | -0.757, 0.233 |
| **Lower** |  |  | 1.312 | -3.336, 5.961 |  |  | -0.737 | -4.254, 2.781 |  |  | -0.286 | -0.978, 0.406 |
| **Constant** | 154.9 | 131.7, 178.0 | 154.6 | 131.6, 177.6 | 89.48 | 67.29, 111.7 | 89.42 | 67.34, 111.5 | 8.045 | 2.880, 13.21 | 7.921 | 2.758, 13.08 |
| **Observations** | 4,692 | | 4,692 | | 3,338 | | 3,338 | | 3,498 | | 3,498 | |
| **R2 (Adjusted)** | 0.11 | | 0.11 | | 0.082 | | 0.081 | | 0.086 | | 0.086 | |

TableA.4b SDQ scores, IPW-adjusted.

|  | **(1a)** | | **(1b)** | | **(2a)** | | **(2b)** | | **(3a)** | | **(3b)** | |
| --- | --- | --- | --- | --- | --- | --- | --- | --- | --- | --- | --- | --- |
|  | **MCS4: Total SDQ binary (imputed)**  **Interaction: No** | | **MCS4: Total SDQ binary (imputed)**  **Interaction: Yes** | | **MCS5 Total SDQ binary(imputed)**  **Interaction: No** | | **MCS5 Total SDQ binary(imputed)**  **Interaction: Yes** | | **MCS6 Total SDQ binary(imputed)**  **Interaction: No** | | **MCS6 Total SDQ binary(imputed)**  **Interaction: Yes** | |
|  | OR | ci95 | OR | ci95 | OR | ci95 | OR | ci95 | OR | ci95 | OR | ci95 |
|  |  |  |  |  |  |  |  |  |  |  |  |  |
| **Summer holidays After (Ref: Before)** | 1.270 | 0.969, 1.664 | 1.168 | 0.611, 2.232 | 0.826 | 0.602, 1.133 | 0.948 | 0.440, 2.043 | 1.261 | 0.980, 1.621 | 1.111 | 0.652, 1.894 |
| **Mother’s Education (Ref: Higher)** |  |  |  |  |  |  |  |  |  |  |  |  |
| **Intermediate** | 2.259 | 1.564, 3.262 | 2.252 | 1.631, 3.110 | 1.501 | 0.991, 2.273 | 1.653 | 1.144, 2.389 | 2.105 | 1.548, 2.863 | 2.056 | 1.490, 2.838 |
| **Lower** | 5.158 | 3.299, 8.065 | 4.355 | 2.939, 6.452 | 2.472 | 1.531, 3.993 | 2.521 | 1.571, 4.046 | 3.468 | 2.318, 5.188 | 2.822 | 1.820, 4.374 |
| **Interaction Term (Ref: Higher)** |  |  |  |  |  |  |  |  |  |  |  |  |
| **Intermediate** |  |  | 1.003 | 0.484, 2.080 |  |  | 0.805 | 0.332, 1.950 |  |  | 1.048 | 0.570, 1.924 |
| **Lower** |  |  | 1.360 | 0.586, 3.157 |  |  | 0.948 | 0.351, 2.563 |  |  | 1.494 | 0.698, 3.201 |
| **Constant** | 0.418 | 0.010, 18.30 | 0.411 | 0.010, 17.46 | 0.435 | 0.000, 276.3 | 0.395 | 0.001, 252.6 | 1.085 | 0.002, 501,1 | 1,205 | 0.003, 584.2 |
| **Observations** | 4,670 | | 4,670 | | 3,272 | | 3,272 | | 3,700 | | 3,700 | |
| **Pseudo-R2** | 0.067 | | 0.068 | | 0.036 | | 0.037 | | 0.032 | | 0.033 | |

Table A.4c SDQ prosocial scores, IPW-adjusted.

|  | **(1a)** | | **(1b)** | | **(2a)** | | **(2b)** | | **(3a)** | | **(3b)** | |
| --- | --- | --- | --- | --- | --- | --- | --- | --- | --- | --- | --- | --- |
|  | **MCS4: Prosocial total binary(imputed)** | | **MCS4: Prosocial total binary(imputed)** | | **MCS5: Prosocial total binary(imputed)** | | **MCS5: Prosocial total binary(imputed)** | | **MCS6: Prosocial total binary(imputed)** | | **MCS6: Prosocial total binary(imputed)** | |
|  | OR | ci95 | OR | ci95 | OR | ci95 | OR | ci95 | OR | ci95 | OR | ci95 |
|  |  |  |  |  |  |  |  |  |  |  |  |  |
| **Summer holidays After (Ref: Before)** | 1.066 | 0.729, 1.559 | 1.273 | 0.622, 2.605 | 1.175 | 0.709, 1.948 | 1.588 | 0.447, 5.637 | 1.098 | 0.786, 1.533 | 0.819 | 0.446, 1.503 |
| **Mother’s Education (Ref: Higher)** |  |  |  |  |  |  |  |  |  |  |  |  |
| **Intermediate** | 1.159 | 0.724, 1.854 | 1.178 | 0.785, 1.769 | 1.381 | 0.668, 2.855 | 1.632 | 0.890, 2.990 | 1.468 | 1.027, 2.098 | 1.198 | 0.827, 1.736 |
| **Lower** | 1.249 | 0.698, 2.236 | 1.870 | 1.121, 3.120 | 1.878 | 0.840, 4.197 | 2.352 | 1.072, 5.162 | 2.122 | 1.261, 3.571 | 1.903 | 1.024, 3.536 |
| **Interaction Term**  **Ref: Higher** |  |  |  |  |  |  |  |  |  |  |  |  |
| **Intermediate** |  |  | 0.972 | 0.407, 2.319 |  |  | 0.727 | 0.186, 2.832 |  |  | 1.499 | 0.731, 3.074 |
| **Lower** |  |  | 0.428 | 0.126, 1.457 |  |  | 0.650 | 0.137, 3.085 |  |  | 1.247 | 0.451, 3.453 |
| **Constant** | 0.044 | 0.000, 26.26 | 0.043 | 0.001, 26.09 | 0.182 | 0.000, 1663 | 0.158 | 0.000, 1,208 | 0.004 | 0.000,11.94 | 0.004 | 0.000, 12.00 |
| **Observations** | 4,709 | | 4,709 | | 3,285 | | 3,285 | | 3,703 | | 3,703 | |
| **Pseudo-R2** | 0.019 | | 0.022 | | 0.030 | | 0.031 | | 0.042 | | 0.043 | |

Table A5 Regressions of reading ability, SDQ total difficulties and prosocial scores on income quintile, period, and income quintile by period for children aged 7 (model 1a-b), 11 (model 2a-b) and 14 (model 3a-b)

Table A.5a Word ability scores, IPW-adjusted.

|  | **(1a)** | | **(1b)** | | **(2a)** | | **(2b)** | | **(3a)** | | **(3b)** | |
| --- | --- | --- | --- | --- | --- | --- | --- | --- | --- | --- | --- | --- |
|  | **S4 CM Word Reading Standard Score**  **Interaction: No** | | **S4 CM Word Reading Standard Score**  **Interaction: Yes** | | **S5 DV Verbal Sims standard score**  **Interaction: No** | | **S5 DV Verbal Sims standard score**  **Interaction: Yes** | | **S6: Word activity score out of 20**  **Interaction: No** | | **S6: Word activity score out of 20**  **Interaction: Yes** | |
|  | b | ci95 | b | ci95 | b | ci95 | b | ci95 | b | ci95 | b | ci95 |
| **Summer holidays After (Ref: Before)** | -2.531 | -4.101, -0.962 | -0.303 | -4.076, 3.470 | 0.570 | -0.673, 1.812 | -1.806 | -4.341, 0.730 | -0.127 | -0.355, 0.102 | -0.037 | -0.586, 0.512 |
| **Income (Ref: Lowest quintile)** |  |  |  |  |  |  |  |  |  |  |  |  |
| **Second quintile** | 2.790 | 0.113, 5.467 | 3.149 | 0.776, 5.521 | 3.710 | 1.992, 5.428 | 1.757 | 0.065, 3.449 | 0.054 | -0.336, 0.444 | 0.167 | -0.343, 0.676 |
| **Third quintile** | 6.270 | 3.596, 8.944 | 7.676 | 5.258, 10.09 | 5.252 | 3.430, 7.074 | 4.071 | 2.344, 5.797 | 0.796 | 0.410, 1.182 | 0.620 | 0.111, 1.130 |
| **Fourth quintile** | 8.167 | 5.501, 10.83 | 10.90 | 8.488, 13.32 | 7.263 | 5.087, 9.439 | 5.424 | 3.597, 7.250 | 1.061 | 0.689, 1.433 | 1.311 | 0.819, 1.802 |
| **Highest quintile** | 13.29 | 10.69, 15.89 | 14.36 | 11.93, 16.79 | 8.684 | 6.958, 10.41 | 7.380 | 5.637, 9.122 | 1.759 | 1.375, 2.144 | 1.805 | 1.313, 2.298 |
| **Interaction Term**  **Ref: Lowest quintile** |  |  |  |  |  |  |  |  |  |  |  |  |
| **Second quintile** |  |  | -0.728 | -5.992, 4.535 |  |  | 3.855 | 0.457, 7.254 |  |  | -0.216 | -0.973, 0.541 |
| **Third quintile** |  |  | -2.814 | -8.088, 2.459 |  |  | 2.283 | -1.202, 5.769 |  |  | 0.346 | -0.390, 1.083 |
| **Fourth quintile** |  |  | -5.494 | -10.64, -0.349 |  |  | 3.619 | -0.634, 7.871 |  |  | -0.489 | -1.190, 0.212 |
| **Highest quintile** |  |  | -2.125 | -7.059, 2.808 |  |  | 2.546 | -0.795, 5.888 |  |  | -0.086 | -0.807, 0.635 |
| **Constant** | 139.0 | 116.0, 162.0 | 138.6 | 115.4, 161.9 | 78.91 | 55.98, 101.8 | 79.92 | 57.14, 102.7 | 6.237 | 1.053, 11.42 | 6.487 | 1.382, 11.59 |
| **Observations** | 4,692 | | 4,692 | | 3,338 | | 3,338 | | 3,498 | | 3,498 | |
| **R2 (Adjusted)** | 0.093 | | 0.095 | | 0.094 | | 0.098 | | 0.069 | | 0.070 | |

Table A.5b SDQ scores, IPW-adjusted.

|  | **(1a)** | | **(1b)** | | **(2a)** | | **(2b)** | | **(3a)** | | **(3b)** | |
| --- | --- | --- | --- | --- | --- | --- | --- | --- | --- | --- | --- | --- |
|  | **MCS4: Total SDQ binary (imputed)**  **Interaction: No** | | **MCS4: Total SDQ binary (imputed)**  **Interaction: Yes** | | **MCS5 Total SDQ binary(imputed)**  **Interaction: No** | | **MCS5 Total SDQ binary(imputed)**  **Interaction: Yes** | | **MCS6 Total SDQ binary(imputed)**  **Interaction: No** | | **MCS6 Total SDQ binary(imputed)**  **Interaction: Yes** | |
|  | OR | ci95 | OR | ci95 | OR | ci95 | OR | ci95 | OR | ci95 | OR | ci95 |
| **Summer holidays After (Ref: Before)** | 1.290 | 0.978, 1.702 | 1.149 | 0.676, 1.952 | 0.819 | 0.597, 1.124 | 1.054 | 0.615, 1.809 | 1.275 | 0.990, 1.641 | 1.278 | 0.768, 2.124 |
| **Income (Ref: Lowest quintile)** |  |  |  |  |  |  |  |  |  |  |  |  |
| **Second quintile** | 0.853 | 0.580, 1.255 | 0.772 | 0.553, 1.076 | 0.788 | 0.514, 1.207 | 0.977 | 0.633, 1.507 | 0.604 | 0.415, 0.879 | 0.587 | 0.380, 0.907 |
| **Third quintile** | 0.562 | 0.363, 0.871 | 0.491 | 0.342, 0.703 | 0.455 | 0.299, 0.692 | 0.515 | 0.324, 0.820 | 0.408 | 0.275, 0.607 | 0.437 | 0.281, 0.680 |
| **Fourth quintile** | 0.416 | 0.263, 0.657 | 0.325 | 0.216, 0.490 | 0.274 | 0.168, 0.447 | 0.427 | 0.257, 0.710 | 0.221 | 0.145, 0.337 | 0.253 | 0.159, 0.400 |
| **Highest quintile** | 0.210 | 0.129, 0.343 | 0.271 | 0.180, 0.408 | 0.347 | 0.196, 0.612 | 0.311 | 0.186, 0.520 | 0.188 | 0.125, 0.284 | 0.150 | 0.094, 0.239 |
| **Interaction Term**  **Ref: Lowest quintile** |  |  |  |  |  |  |  |  |  |  |  |  |
| **Second quintile** |  |  | 1.209 | 0.580, 2.522 |  |  | 0.642 | 0.263, 1.566 |  |  | 1.057 | 0.511, 2.186 |
| **Third quintile** |  |  | 1.287 | 0.571, 2.903 |  |  | 0.782 | 0.334, 1.835 |  |  | 0.877 | 0.425, 1.810 |
| **Fourth quintile** |  |  | 1.568 | 0.679, 3.620 |  |  | 0.324 | 0.094, 1.119 |  |  | 0.769 | 0.347, 1.705 |
| **Highest quintile** |  |  | 0.578 | 0.208, 1.601 |  |  | 1.290 | 0.423, 3.933 |  |  | 1.494 | 0.712, 3.136 |
| **Constant** | 2.358 | 0.044,127.02 | 2.280 | 0.043,123.80 | 1.611 | 0.002, 1,121 | 1.610 | 0.002, 1,039 | 9.843 | 0.017, 5,479 | 9.10 | 0.017, 4,958 |
| **Observations** | 4,670 | | 4,670 | | 3,272 | | 3,272 | | 3,700 | | 3,700 | |
| **Pseudo-R2** | 0.066 | | 0.069 | | 0.057 | | 0.060 | | 0.060 | | 0.062 | |

Table A.5c SDQ prosocial scores, IPW-adjusted.

|  | **(1a)** | | **(1b)** | | **(2a)** | | **(2b)** | | **(3a)** | | **(3b)** | |
| --- | --- | --- | --- | --- | --- | --- | --- | --- | --- | --- | --- | --- |
|  | **MCS4: Prosocial total binary(imputed)** | | **MCS4: Prosocial total binary(imputed)** | | **MCS5: Prosocial total binary(imputed)** | | **MCS5: Prosocial total binary(imputed)** | | **MCS6: Prosocial total binary(imputed)** | | **MCS6: Prosocial total binary(imputed)** | |
|  | OR | ci95 | OR | ci95 | OR | ci95 | OR | ci95 | OR | ci95 | 0R | ci95 |
| **Summer holidays After (Ref: Before)** | 1.070 | 0.731, 1.565 | 1.246 | 0.596, 2.606 | 1.167 | 0.703, 1.936 | 1.075 | 0.490, 2.359 | 1.095 | 0.785, 1.527 | 0.973 | 0.492, 1.926 |
| **Income (Ref: Lowest quintile)** |  |  |  |  |  |  |  |  |  |  |  |  |
| **Second quintile** | 0.689 | 0.403, 1.179 | 0.844 | 0.519, 1.372 | 0.569 | 0.302, 1.071 | 0.460 | 0.225, 0.942 | 0.390 | 0.225, 0.678 | 0.422 | 0.219, 0.813 |
| **Third quintile** | 0.575 | 0.299, 1.106 | 0.664 | 0.396, 1.116 | 0.268 | 0.146, 0.491 | 0.378 | 0.178, 0.805 | 0.342 | 0.210, 0.557 | 0.370 | 0.211, 0.649 |
| **Fourth quintile** | 0.665 | 0.367, 1.206 | 0.627 | 0.365, 1.079 | 0.258 | 0.112, 0.592 | 0.245 | 0.104, 0.578 | 0.305 | 0.182, 0.510 | 0.248 | 0.137, 0.448 |
| **Highest quintile** | 0.421 | 0.231, 0.769 | 0.492 | 0.276, 0.876 | 0.385 | 0.164, 0.905 | 0.289 | 0.129, 0.650 | 0.335 | 0.207, 0.541 | 0.232 | 0.134, 0.403 |
| **Interaction Term**  **Ref: Lowest quintile** |  |  |  |  |  |  |  |  |  |  |  |  |
| **Second quintile** |  |  | 0.671 | 0.232, 1.939 |  |  | 1.456 | 0.431, 4.912 |  |  | 0.829 | 0.256, 2.684 |
| **Third quintile** |  |  | 0.759 | 0.218, 2.645 |  |  | 0.394 | 0.109, 1.424 |  |  | 0.856 | 0.322, 2.277 |
| **Fourth quintile** |  |  | 1.128 | 0.376, 3.388 |  |  | 1.093 | 0.216, 5.543 |  |  | 1.466 | 0.557, 3.860 |
| **Highest quintile** |  |  | 0.746 | 0.236, 2.356 |  |  | 1.716 | 0.353, 8.356 |  |  | 1.914 | 0.777, 4.712 |
| **Constant** | 0.105 | 0.001, 58.96 | 0.105 | 0.001, 57.87 | 0.822 | 0.000, 5,945 | 0.912 | 0.002, 7,782 | 0.012 | 0.000, 44.33 | 0.012 | 0.000, 46,31 |
| **Observations** | 4,709 | | 4,709 | | 3,285 | | 3,285 | | 3,703 | | 3,703 | |
| **Pseudo-R2** | 0.028 | | 0.029 | | 0.057 | | 0.061 | | 0.062 | | 0.066 | |

Table A6 Regressions of reading ability, SDQ total difficulties and prosocial scores on neighbourhood deprivation, period, and neighbourhood deprivation (IMD) by period for children aged 7 (model 1a-b), 11 (model 2a-b) and 14 (model 3a-b)

Table A.6a Word ability Scores IPW-adjusted.

|  | **(1a)** | | **(1b)** | | **(2a)** | | **(2b)** | | **(3a)** | | **(3b)** | |
| --- | --- | --- | --- | --- | --- | --- | --- | --- | --- | --- | --- | --- |
|  | **S4 CM Word Reading Standard Score**  **Interaction: No** | | **S4 CM Word Reading Standard Score**  **Interaction: Yes** | | **S5 DV Verbal Sims standard score**  **Interaction: No** | | **S5 DV Verbal Sims standard score**  **Interaction: Yes** | | **S6: Word activity score out of 20**  **Interaction: No** | | **S6: Word activity score out of 20**  **Interaction: Yes** | |
|  | b | ci95 | b | ci95 | b | ci95 | b | ci95 | b | ci95 | b | ci95 |
| **Summer holidays After (Ref: Before)** | -2.474 | -4.078, -0.870 | -3.730 | -8.467, 1.008 | 0.559 | -0.728, 1.846 | 2.848 | -0.942, 6.639 | -0.135 | -0.359, 0.090 | -0.309 | -0.962, 0.343 |
| **Most Deprived Decile** |  |  |  |  |  |  |  |  |  |  |  |  |
| **10 - < 20%** | -1.489 | -4.823, 1.844 | -3.105 | -6.051, -0.160 | -0.620 | -3.270, 2.029 | 1.623 | -0.454, 3.700 | 0.635 | 0.203, 1.068 | 0.379 | -0.152, 0.911 |
| **20 - < 30%** | -2.233 | -5.672, 1.206 | -1.450 | -4.555, 1.655 | -1.030 | -3.774, 1.714 | -1.204 | -3.447, 1.040 | 0.366 | -0.12, 0.854 | 0.704 | 0.106, 1.302 |
| **30 - < 40%** | -0.404 | -4.101, 3.293 | -3.231 | -6.400, -0.061 | -1.518 | -4.606, 1.571 | -0.898 | -3.294, 1.499 | 1.002 | 0.547, 1.457 | 0.705 | 0.079, 1.331 |
| **40 - < 50%** | -0.785 | -4.423, 2.852 | -4.090 | -7.332, -0.848 | -1.436 | -4.073, 1.201 | -0.0456 | -2.337, 2.246 | 0.972 | 0.526, 1.418 | 0.877 | 0.269, 1.485 |
| **50 - < 60%** | -0.757 | -4.251, 2.737 | -1.817 | -5.140, 1.506 | -1.173 | -3.564, 1.218 | -0.885 | -3.210, 1.440 | 1.107 | 0.601, 1.613 | 0.954 | 0.329, 1.579 |
| **60 - < 70%** | -3.536 | -7.109, 0.037 | -2.179 | -5.270, 0.911 | 0.213 | -2.269, 2.695 | 2.385 | 0.441, 4.329 | 1.193 | 0.720, 1.666 | 1.312 | 0.660, 1.964 |
| **70 - < 80%** | 1.351 | -2.200, 4.902 | -0.021 | -3.071, 3.030 | -0.914 | -3.597, 1.768 | 1.393 | -0.590, 3.376 | 1.619 | 1.171, 2.067 | 1.467 | 0.861, 2.074 |
| **80 - < 90%** | 1.133 | -2.434, 4.701 | 1.838 | -1.117, 4.792 | -1.818 | -4.727, 1.090 | 0.375 | -1.995, 2.745 | 1.933 | 1.365, 2.502 | 1.573 | 0.943, 2.202 |
| **Least Deprived Decile** | 0.309 | -3.203, 3.821 | 1.373 | -1.646, 4.392 | 2.795 | 0.507, 5.083 | 1.770 | -0.350, 3.890 | 2.122 | 1.609, 2.634 | 2.094 | 1.526, 2.662 |
| **Interaction Term**  **Ref: Most Deprived Decile** |  |  |  |  |  |  |  |  |  |  |  |  |
| **10 - < 20%** |  |  | 3.170 | -3.405, 9.745 |  |  | -4.449 | -9.610, 0.712 |  |  | 0.491 | -0.365, 1.346 |
| **20 - < 30%** |  |  | -1.630 | -8.417, 5.157 |  |  | 0.285 | -5.048, 5.618 |  |  | -0.593 | -1.531, 0.345 |
| **30 - < 40%** |  |  | 5.892 | -1.442, 13.23 |  |  | -1.348 | -7.472, 4.777 |  |  | 0.572 | -0.342, 1.487 |
| **40 - < 50%** |  |  | 6.472 | -0.611, 13.56 |  |  | -2.897 | -8.062, 2.268 |  |  | 0.186 | -0.714, 1.086 |
| **50 - < 60%** |  |  | 2.094 | -4.876, 9.065 |  |  | -0.704 | -5.442, 4.034 |  |  | 0.302 | -0.706, 1.311 |
| **60 - < 70%** |  |  | -2.857 | -9.901, 4.187 |  |  | -4.645 | -9.637, 0.348 |  |  | -0.249 | -1.202, 0.705 |
| **70 - < 80%** |  |  | 2.737 | -4.416, 9.890 |  |  | -4.815 | -10.04, 0.411 |  |  | 0.294 | -0.598, 1.187 |
| **80 - < 90%** |  |  | -1.390 | -8.286, 5.506 |  |  | -4.644 | -10.38, 1.091 |  |  | 0.701 | -0.410, 1.812 |
| **Least Deprived Decile** |  |  | -2.233 | -9.138, 4.673 |  |  | 2.546 | -1.864, 6.956 |  |  | 0.0512 | -0.975, 1.077 |
| **Constant** | 151.8 | 127.7, 175.9 | 152.8 | 128.6, 176.9 | 86.79 | 63.14, 110.4 | 87.01 | 64.05, 110.0 | 5.282 | 0.135, 10.43 | 5.447 | 0.330, 10.56 |
| **Observations** | 4,692 | | 4,692 | | 3,338 | | 3,338 |  | 3,498 |  | 3,498 |  |
| **R2 (Adjusted)** | 0.036 | | 0.042 | | 0.025 | | 0.037 |  | 0.068 |  | 0.071 |  |

Table A.6b SDQ scores, IPW-adjusted.

|  | **(1a)** | | **(1b)** | | **(2a)** | | **(2b)** | | **(3a)** | | **(3b)** | |
| --- | --- | --- | --- | --- | --- | --- | --- | --- | --- | --- | --- | --- |
|  | **MCS4: Total SDQ binary (imputed) Interaction: No** | | **MCS4: Total SDQ binary (imputed)**  **Interaction: Yes** | | **MCS5 Total SDQ binary(imputed)**  **Interaction: No** | | **MCS5 Total SDQ binary(imputed)**  **Interaction: Yes** | | **MCS6 Total SDQ binary(imputed) Interaction: No** | | **MCS6 Total SDQ binary(imputed) Interaction: Yes** | |
|  | OR | ci95 | OR | ci95 | OR | ci95 | OR | ci95 | OR | ci95 | OR | ci95 |
| **Summer holidays After (Ref: Before)** | 1.272 | 0.967, 1.674 | 1.222 | 0.606, 2.466 | 0.853 | 0.621, 1.172 | 1.482 | 0.590, 3.723 | 1.260 | 0.981, 1.617 | 1.600 | 0.886, 2.889 |
| **Most Deprived Decile** |  |  |  |  |  |  |  |  |  |  |  |  |
| **10 - < 20%** | 0.761 | 0.455, 1.271 | 0.995 | 0.623, 1.591 | 0.470 | 0.250, 0.883 | 1.011 | 0.485, 2.108 | 0.733 | 0.474, 1.133 | 0.890 | 0.541, 1.464 |
| **20 - < 30%** | 1.049 | 0.606, 1.814 | 1.008 | 0.625, 1.627 | 0.987 | 0.524, 1.857 | 1.340 | 0.648, 2.771 | 0.765 | 0.483, 1.213 | 0.854 | 0.506, 1.441 |
| **30 - < 40%** | 0.785 | 0.434, 1.420 | 0.696 | 0.423, 1.145 | 1.113 | 0.584, 2.122 | 0.976 | 0.476, 2.001 | 0.521 | 0.321, 0.846 | 0.714 | 0.421, 1.211 |
| **40 - < 50%** | 0.869 | 0.475, 1.589 | 0.737 | 0.447, 1.215 | 0.905 | 0.432, 1.894 | 0.994 | 0.490, 2.016 | 0.566 | 0.353, 0.909 | 0.561 | 0.323, 0.971 |
| **50 - < 60%** | 0.452 | 0.257, 0.792 | 0.757 | 0.457, 1.256 | 0.801 | 0.383, 1.677 | 0.801 | 0.374, 1.718 | 0.623 | 0.381, 1.018 | 0.606 | 0.341, 1.079 |
| **60 - < 70%** | 1.068 | 0.612, 1.865 | 0.825 | 0.500, 1.363 | 0.806 | 0.400, 1.625 | 0.716 | 0.352, 1.457 | 0.411 | 0.235, 0.721 | 0.531 | 0.264, 1.069 |
| **70 - < 80%** | 0.875 | 0.467, 1.636 | 0.739 | 0.450, 1.214 | 0.736 | 0.406, 1.334 | 1.271 | 0.650, 2.485 | 0.386 | 0.236, 0.630 | 0.478 | 0.262, 0.873 |
| **80 - < 90%** | 0.693 | 0.356, 1.349 | 0.509 | 0.297, 0.873 | 0.362 | 0.190, 0.689 | 0.865 | 0.426, 1.757 | 0.416 | 0.225, 0.766 | 0.463 | 0.219, 0.982 |
| **Least Deprived Decile** | 0.577 | 0.322, 1.032 | 0.601 | 0.356, 1.014 | 0.771 | 0.423, 1.407 | 1.274 | 0.655, 2.477 | 0.264 | 0.150, 0.464 | 0.297 | 0.161, 0.551 |
| **Interaction Term**  **Ref: Most Deprived Decile** |  |  |  |  |  |  |  |  |  |  |  |  |
| **10 - < 20%** |  |  | 0.583 | 0.212, 1.599 |  |  | 0.161 | 0.044, 0.590 |  |  | 0.686 | 0.291, 1.618 |
| **20 - < 30%** |  |  | 1.070 | 0.384, 2.979 |  |  | 0.535 | 0.149, 1.919 |  |  | 0.807 | 0.324, 2.005 |
| **30 - < 40%** |  |  | 1.254 | 0.418, 3.764 |  |  | 1.237 | 0.352, 4.355 |  |  | 0.535 | 0.195, 1.463 |
| **40 - < 50%** |  |  | 1.345 | 0.445, 4.068 |  |  | 0.828 | 0.197, 3.482 |  |  | 1.005 | 0.399, 2.531 |
| **50 - < 60%** |  |  | 0.240 | 0.057, 1.020 |  |  | 1.019 | 0.245, 4.233 |  |  | 1.041 | 0.401, 2.705 |
| **60 - < 70%** |  |  | 1.589 | 0.574, 4.402 |  |  | 1.195 | 0.317, 4.513 |  |  | 0.601 | 0.191, 1.884 |
| **70 - < 80%** |  |  | 1.362 | 0.430, 4.314 |  |  | 0.293 | 0.085, 1.010 |  |  | 0.659 | 0.249, 1.747 |
| **80 - < 90%** |  |  | 1.697 | 0.522, 5.516 |  |  | 0.0453 | 0.001, 0.216 |  |  | 0.807 | 0.241, 2.701 |
| **Least Deprived Decile** |  |  | 0.919 | 0.297, 2.845 |  |  | 0.270 | 0.063, 1.163 |  |  | 0.793 | 0.261, 2.405 |
| **Constant** | 0.717 | 0.025,33.73 | 0.867 | 0.017,43.21 | 1.601 | 0.003,888 | 2.237 | 0.005, 1,028 | 5.133 | 0.011, 2,488 | 4.961 | 0.010, 2,344 |
| **Observations** | 4,670 | | 4,670 | | 3,272 | | 3,272 | | 3,700 | | 3,700 | |
| **Pseudo-R2** | 0.037 | | 0.045 | | 0.037 | | 0.060 | | 0.029 | | 0.031 | |

Table A.6c SDQ prosocial scores, IPW-adjusted.

|  | **(1a)** | | **(1b)** | | **(2a)** | | **(2b)** | | **(3a)** | | **(3b)** | |
| --- | --- | --- | --- | --- | --- | --- | --- | --- | --- | --- | --- | --- |
|  | **MCS4: Prosocial total binary(imputed)**  **Interaction: No** | | **MCS4: Prosocial total binary(imputed)**  **Interaction: Yes** | | **MCS5: Prosocial total binary(imputed)**  **Interaction: No** | | **MCS5: Prosocial total binary(imputed) Interaction: Yes** | | **MCS6: Prosocial total binary(imputed)**  **Interaction: No** | | **MCS6: Prosocial total binary(imputed)**  **Interaction: Yes** | |
|  | OR | ci95 | OR | ci95 | OR | ci95 | OR | ci95 | OR | ci95 | OR | ci95 |
| **Summer holidays After (Ref: Before)** | 1.068 | 0.731, 1.559 | 1.678 | 0.603, 4.673 | 1.208 | 0.726, 2.009 | 0.669 | 0.170, 2.639 | 1.093 | 0.782, 1.527 | 1.971 | 0.900, 4.318 |
| **Most Deprived Decile** |  |  |  |  |  |  |  |  |  |  |  |  |
| **10 - < 20%** | 0.785 | 0.353, 1.748 | 1.280 | 0.593, 2.765 | 0.369 | 0.138, 0.991 | 0.250 | 0.080, 0.781 | 1.096 | 0.589, 2.038 | 1.773 | 0.849, 3.702 |
| **20 - < 30%** | 0.722 | 0.326, 1.601 | 1.180 | 0.559, 2.493 | 0.836 | 0.328, 2.132 | 0.689 | 0.227, 2.094 | 1.206 | 0.644, 2.257 | 1.864 | 0.898, 3.871 |
| **30 - < 40%** | 0.567 | 0.259, 1.245 | 0.865 | 0.391, 1.913 | 0.724 | 0.275, 1.905 | 0.507 | 0.168, 1.528 | 0.533 | 0.277, 1.028 | 0.731 | 0.347, 1.542 |
| **40 - < 50%** | 0.733 | 0.318, 1.693 | 0.933 | 0.440, 1.979 | 0.475 | 0.188, 1.199 | 0.616 | 0.205, 1.855 | 0.744 | 0.405, 1.366 | 1.181 | 0.602, 2.317 |
| **50 - < 60%** | 0.804 | 0.356, 1.815 | 0.797 | 0.347, 1.829 | 0.643 | 0.191, 2.165 | 0.102 | 0.022, 0.484 | 0.539 | 0.265, 1.095 | 0.876 | 0.415, 1.845 |
| **60 - < 70%** | 0.738 | 0.335, 1.628 | 1.076 | 0.492, 2.353 | 0.718 | 0.264, 1.947 | 0.614 | 0.219, 1.725 | 0.658 | 0.294, 1.470 | 0.501 | 0.221, 1.133 |
| **70 - < 80%** | 0.499 | 0.210, 1.185 | 0.879 | 0.394, 1.962 | 0.648 | 0.249, 1.685 | 0.353 | 0.110, 1.134 | 0.473 | 0.251, 0.890 | 0.674 | 0.305, 1.486 |
| **80 - < 90%** | 0.522 | 0.215, 1.267 | 0.890 | 0.404, 1.962 | 0.234 | 0.082, 0.668 | 0.157 | 0.049, 0.508 | 0.760 | 0.375, 1.541 | 1.339 | 0.558, 3.211 |
| **Least Deprived Decile** | 0.982 | 0.414, 2.329 | 0.609 | 0.267, 1.388 | 0.610 | 0.217, 1.712 | 0.484 | 0.164, 1.424 | 0.489 | 0.252, 0.950 | 0.590 | 0.290, 1.202 |
| **Interaction Term**  **Ref: Most Deprived Decile** |  |  |  |  |  |  |  |  |  |  |  |  |
| **10 - < 20%** |  |  | 0.394 | 0.073, 2.120 |  |  | 2.185 | 0.327, 14.59 |  |  | 0.410 | 0.126, 1.339 |
| **20 - < 30%** |  |  | 0.390 | 0.080, 1.905 |  |  | 1.560 | 0.238, 10.24 |  |  | 0.436 | 0.126, 1.515 |
| **30 - < 40%** |  |  | 0.445 | 0.096, 2.069 |  |  | 2.078 | 0.314, 13.75 |  |  | 0.566 | 0.161, 1.992 |
| **40 - < 50%** |  |  | 0.661 | 0.138, 3.165 |  |  | 0.413 | 0.061, 2.813 |  |  | 0.414 | 0.123, 1.402 |
| **50 - < 60%** |  |  | 1.042 | 0.238, 4.568 |  |  | 16.32 | 1.745, 152.7 |  |  | 0.397 | 0.094, 1.676 |
| **60 - < 70%** |  |  | 0.503 | 0.112, 2.251 |  |  | 1.426 | 0.182, 11.20 |  |  | 1.464 | 0.374, 5.725 |
| **70 - < 80%** |  |  | 0.306 | 0.048, 1.929 |  |  | 3.187 | 0.496, 20.48 |  |  | 0.523 | 0.154, 1.781 |
| **80 - < 90%** |  |  | 0.351 | 0.056, 2.339 |  |  | 2.236 | 0.300, 16.64 |  |  | 0.339 | 0.085, 1.348 |
| **Least Deprived Decile** |  |  | 2.145 | 0.495, 9.298 |  |  | 1.607 | 0.160, 16.10 |  |  | 0.717 | 0.208, 2.472 |
| **Constant** | 0.073 | 0.000,47.86 | 0.042 | 0.001,31.82 | 0.810 | 0.000, 5,136 | 1,881 | 0.00, 20,076 | 0.008 | 0.000,26.20 | 0.004 | 0.000,10.26 |
| **Observations** | 4,709 | | 4,709 | | 3,285 | | 3,285 |  | 3,703 | | 3,703 | |
| **Pseudo-R2** | 0.024 | | 0.035 | | 0.040 | | 0.057 |  | 0.050 | | 0.056 | |
